# Evaluation of Cause of Deaths' Validity Using Outcome Measures from a Prospective, Population Based Cohort Study in Tehran, Iran

**DOI:** 10.1371/journal.pone.0031427

**Published:** 2012-02-15

**Authors:** Davood Khalili, Alireza Mosavi-Jarrahi, Fatemeh Eskandari, Yasaman Mousavi-Jarrahi, Farzad Hadaegh, Mohammadali Mohagheghi, Fereidoun Azizi

**Affiliations:** 1 Prevention of Metabolic Disorders Research Center, Research Institute for Endocrine Sciences, Shahid Beheshti University of Medical Sciences, Tehran, Iran; 2 Department of Epidemiology, School of Public Health, Shahid Beheshti University of Medical Sciences, Tehran, Iran; 3 The Health Metrics Research Center, Department of Social Medicine and Public Health, Medical School, Shahid Beheshti University of Medical Sciences, Tehran, Iran; 4 The Cancer Research Center of the Cancer Institute, Tehran University of Medical Sciences, Tehran, Iran; Uniformed Services University of Health Sciences, United States of America

## Abstract

**Objective:**

The aim of this study was to evaluate the validity of cause of death stated in death certificates in Tehran using outcome measures of the Tehran Lipid and Glucose Study (TLGS), an ongoing prospective cohort study.

**Methods:**

The cohort was established in 1999 in a population of 15005 people, 3 years old and over, living in Tehran; 3551 individuals were added to this population three years later. As part of cohort's outcome measures, deaths occurring in the cohort are investigated by a panel of medical specialists (Cohort Outcome Panel-COP) and underlying cause of death is determined for each death. The cause of death assigned in a deceased's original death certificate was evaluated against the cause of death determined by COP and sensitivity and positive predictive values (PPV) were determined. In addition, determinants of assigning accurate underlying cause of death were determined using logistic regression model.

**Result:**

A total of 231 death certificates were evaluated. The original death certificates over reported deaths due to neoplasms and underreported death due to circulatory system and transport accidents. Neoplasms with sensitivity of 0.91 and PPV of 0.71 were the most valid category. The disease of circulatory system showed moderate degree of validity with sensitivity of 0.67 and PPV of 0.78. The result of logistic regression indicated if the death certificate is issued by a general practitioner, there is 2.3 (95% CI 1.1, 5.1) times chance of being misclassified compared with when it is issued by a specialist. If the deceased is more than 60 years, the chance of misclassification would be 2.5 times (95% CI of 1.1, 5.9) compared with when the deceased is less than 60 years.

## Introduction

Mortality data have been one of the oldest information available to the health authorities and have been utilized to monitor the health of different communities since early 17th century when the first death registration was established in England and Wales [1]. Parallel to the utilization of mortality data, concern on the validity of cause of death had been a long lasting challenge to epidemiologist who bear the task of interpreting the mortality information at the community level [2]. The validity challenges of mortality data originate from the very fact that assigning an underlying cause of death from chain of events ending to death is more of opinion nature than an objective and well defined procedure [1]. While there are enormous materials and guidelines as well as recommendations on how to accurately assign an underlying cause of death however, it is established that if the autopsy is not the means to determine the underlying cause, the misclassification of cause of death is a serious problem in reporting of mortality data. The degree of misclassification is influenced by several factors including; the true underlying cause of death, the mechanism that judgment on cause of death is based on (clinical information, autopsy report, or just simple inquiry from the next of kin of the deceased), the age at which death occurs, and lack of a standard procedures and quality controls in management of death certificate at community level [3]–[7]. Studies of accuracy and validity of underlying cause of death have reported different degrees of accuracy for different casual categories [7]. While cancer is one the underlying cause that enjoys acceptable degree of accuracy [8], the cardiovascular diseases as leading cause of death suffers a great deal of inaccuracy [9]. Studies of assessing validity and accuracy of cause of death in different communities have employed several methodology among them the sensitivity analysis of issued cause of death against a gold standard such as autopsy reports or reassessment of underling cause by either re-abstracting of medical information of the deceased or application of standardized structured questionnaire in the form of verbal autopsy [10], [11]. The present study examines the validity of underlying cause of death as stated in death certificate for group of deceased who were registered members of a prospective cohort that were monitored for several exposures and outcomes (both morbidity and mortality) in a urban population in Tehran, Iran.

## Materials and Methods

This study was approved by the ethical committee of the Research Institute for Endocrine Sciences and the ethical committee of the Cancer Institute Research Center.

The Tehran Lipid and Glucose Study (TLGS) is an ongoing prospective cohort study of 18556 people, age over three years, living in the Tehran, a mega city located in the central plateau of Iran, 1500 kilometers north of the Persian Gulf. The cohort was established in 1999 with a population of 15005 and three years later, 3551 members were added to this number. The cohort composed of a randomly selected households residing in the district number 13 of the Tehran metropolis. Demographic, medical history, physical examination and laboratory data were collected at baseline (entry into the cohort) for each cohort member and repeated every three years. As part of cohort procedural's data collection, trained nurses contacted cohort member every year and measured all the medical events experienced by the cohort member during the year. Any reported event was followed a home visit by a trained physician and collection of medical data (diagnostic, or treatment) from hospital or other service providers. Detailed methodology of cohort has been published in details elsewhere [12], [13]. If a death happened to a member, a verbal autopsy was performed using a standard questionnaire. The questionnaire consists of questions about time and location (in hospital or home) of death plus medical events and complications leading to death. Medical data were collected for each deceased person by referring to medical record departments of service providers (hospital or outpatient). The collected data were evaluated by a panel of specialist called the Cohort Outcome Panel (COP) which included internist, endocrinologist, cardiologist, and epidemiologist. The panel assigned an underlying cause of death for each deceased member.

For the purpose of this study, information about all members registered at baseline and deceased during the follow-up were obtained from the cohort's managing office. The original death certificates (the certificate that was legally issued for burial) for the deceased were sought from two sources, the Tehran municipality morgue office, and the next of kin of the deceased. In Iran, according to legal requirement, for disposal a deceased, a death certificate is needed and without an official death certificate, the morgue office cannot allow burial and the case is referred to the legal inquiry. Just a registered medical doctor can issue the death certificate. The Tehran municipality morgue requires an original copy of the death certificate for the disposal of the deceased and the office keep the copy as part of their paperwork and documentations. The structure of the death certificates in Iran follows the basic requirement of the World Health Organization (WHO) recommendations which includes at least two categories of cause of death; an underlying, and a mode of death. The health authorities have recently modified the death certificate and enhanced its contents, but not all the medical doctors or hospitals use the enhanced one, leaving two different formats of the death certificates available to physicians. The death certificate needs not to be issued by physician caring the patient and it very often happened to be issued by a freelance general practitioner (GP) unless the patient dies in the hospital. If the patient dies in a hospital, the attending physician issues the death certificate. While the health authorities constantly attempt to educate the physicians in preparing an accurate death certificate, no formal education or certification is needed to be able to issue death certificate. In this study, the cause of death declared by the official death certificate was evaluated against the cause of death determined by the cohort outcome panel (COP), considered as the gold standard. For the purpose of this study, both cause-of-death declared in the original death certificate and assigned by the COP were coded to the ICD-10 rubrics by a trained medical technologist. The sensitivity and positive predictive values were determined at two levels of ICD-10 grouping 1) the main disease/organ system grouping category (here after called main category) and 2) disease sub-grouping category, (hereafter called sub-category). Sensitivity was calculated as the proportion of true positives (concordant declaration of cause of death by death certificate and the cohort outcome panel) divided by the sum of true positive and false negative diagnoses (discordant declaration of cause of death by death certificate and the cohort outcome panel), and positive predictive value (PPV) as the number of true positives divided by true positives and false positives. In order to determine what factors correlate with a better accuracy of assigning a cause of death, using a binary outcome of either concordant (if the cause of death stated in the original death certificate was the same as the COP assigned cause of death) or discordant (if the cause of death stated in the original death certificate was different from the COP assigned cause of death) between the stated underlying cause in the original death certificate and the cause determined by the COP , a logistic regression model was fitted to the data to explore the effect of age at time of death (categorized as under 60, and 60 and over), the gender (as male and female), the place of death (in hospital or out of hospital) and the issuing the death certificate by specialist or a general physician. The goodness of fit of the logistic regression was evaluated using Hosmer-Lemeshow Chi-square test. All data analyses were performed using SPSS version 15.0 (SPSS, Inc., Chicago IL).

## Results

A total of 367 deaths occurred for 118994 person years of follow-up with a crude mortality rate of 308 cases per 100,000 person-years. Out of 367 deaths, the original death certificate was retrieved for 231 cases either from the Tehran municipality morgue office or from the next of kin. No death certificate was available for 136 of deaths either due to the fact that the deceased was not buried in the Tehran cemetery, or the death certificate was missing, or the next of kin did not provide the death certificate. Out of 231 death certificates, 120 were issued by a general practitioner, 33 by legal medicine specialist and the resting by other specialists. One hundred fifty seven (68%) cases of the deceased were male and just 74 (32%) cases were female. The distribution of age at death showed 29(13%) cases less than 50 years, 26(11%) between 50–60, and 176 (76%) over 60 years.

The major disease category reported as underlying cause of death for both the COP and the original death certificates were the disease of circulatory system (ICD10 rubric I00-I99), Neoplasms (ICD10 rubric C00-C48), and transport accident (ICD10 rubric V01-X59). The frequency of symptoms and signs without any classified definition (ICD10 rubric R00-R99) was 41 (17.7%) for the original death certificates and undefined cases or cases with insufficient data were 42 (18.2) for the COP assigned cause. Table 1 presents the frequency distribution of the underlying causes of death based on the major category of ICD10 for both, the original death certificate and the assigned cause by the COP.

**Table 1:** The frequency distribution of underlying cause of death for both death certificates and Cohort outcome panel.

Disease category grouping (ICD-10 rubric)	Underlying cause of death determined by	
	Death Certificate*	Cohort Outcome Panel*
Infectious diseases (A00-B99)	3 (1.3)	0 (0.0)
Neoplasms (C00-D48)	45 (19.5)	35 (15.2)
Endocrine and metabolic diseases (E00-E88)	5 (2.2)	1 (0.4)
Mental and behavioral disorders (F00-F99)	0 (0.0)	1 (0.4)
Diseases of the nervous system (G00-G98)	4 (1.7)	5 (2.2)
Diseases of the circulatory system (I00-I99)	90 (39.0)	103 (44.6)
Diseases of the respiratory system (J00-J98)	9 (3.9)	5 (2.2)
Diseases of the digestive system (K00-K92)	8 (3.5)	8 (3.5)
Diseases of the skin and subcutaneous tissue (L00-L98)	1(0.4)	0 (0.0)
The musculoskeletal system and connective tissue (M00-M99_)	1 (0.4)	1 (0.4)
Diseases of the genitourinary system (N00-N98)	1 (0.4)	4 (1.7)
Symptoms and signs not elsewhere classified (R00-R99)	41 (17.7)	0 (0.0)
Transport accidents (V01-X59)	19 (8.2)	22 (9.5)
Legal intervention and operations of war (X85-Y36)	1 (0.4)	3 (1.3)
Intentional self-harm (X60-X86)	0 (0.0)	1 (0.4)
Undefined or insufficient data	3 (1.3)	42 (18.2)
Total	231 (100)	231 (100)

*Expressed as number (%).

Table 2 shows the misclassification matrix for leading causes. For each disease at the major categories, the rows of the matrix indicate the number of deaths that the cause of death has been assigned by COP, while the columns show to which cause each of these deaths was assigned in the original death certificate. In the neoplasm category (C00-D48), out of 45 reported cases by the death certificate, 13 cases were misclassified as neoplasm (a over diagnosis of neoplasm). The COP classified 7 of these as cases with insufficient data, 2 as circulatory system, and resting to other categories (table 2). The other major category with significant number of misclassifications was the disease of circulatory system (I00-I99). While there was 103 cases being truly belong to this category (COP's finding), the original death certificates misclassified 22 cases in the symptoms and signs not elsewhere classified (R00-R99), indicating a under reporting of this category. In the transport accident category (V01-X99), there were 22 truly diagnosed by the COP while the death certificate reports just 19 (a underreporting of the cases in this category). The misclassified in this category were mainly assigned to circulatory system and symptoms and signs (table 2). All the 3 cases declared in the infectious disease category (A00-B99) by the original death certificate were classified to other category by the panel, indicating an over-reporting for this category in the original death certificate. The four cases diagnosed as disease of genitourinary system (N00-N98) by COP, were misclassified by original death certificate to infectious diseases, diseases of the circulatory system or not classified symptoms and signs, indicating an under-reporting.

**Table 2:** Misclassification matrix (expressed as number) for major grouping of ICD10*.

	Death Certificate assignment								
TLGS assignment	NeoplasmsC00-D48	EndocrineE00-E88	Nervous systemG00-G98	Circulatory systemI00-I99	Respiratory systemJ00-J98	Digestive systemK00-K92	Symptoms & signsR00-R99	Transport accidentsV01-X59	Undefined or insufficient data
Neoplasms, C00-D48	32	0	0	1	0	0	2	0	0
Endocrine, E00-E88	0	1	0	0	0	0	0	0	0
Nervous system, G00-G98	1	0	2	0	0	0	1	0	0
Circulatory system, I00-I99	2	1	2	69	3	0	22	0	1
Respiratory system, J00-J98	1	0	0	1	3	0	0	0	0
Digestive system, K00-K92	0	0	0	0	0	6	2	0	0
Symptoms & signs, R00-R99	0	0	0	0	0	0	0	0	0
Transport accidents, V01-X59	0	0	0	4	0	0	2	16	0
Undefined or insufficient data	7	3	0	13	3	2	11	1	1

*Categories with less than 5 events were eliminated.

Assessing the indicators of validity for the underlying cause of death declared in the death certificates and considering the frequency of occurrence, neoplasms with a sensitivity of 0.91 and positive predictive value of 0.71 had the highest indicator of validity. The diseases of circulatory system with sensitivity of 0.67 and positive predictive value of 0.78 were among the frequent underlying cause with relatively moderate degree of validity (table 3 presents the detailed indicators of validity for the main category of ICD10 grouping). Assessing validity based on further sub-categories of ICD10 indicated higher sensitivity among the certain cancers (stomach, pancreas and breast). Among the diseases of the circulatory system in the subcategory of ICD10, the ischemic heart disease (ICD10 rubric I20–I25) and cerebrovascular diseases (ICD10 rubric I60–I69) with sensitivity of 0.57 showed moderate-to-low indicators of validity. (Table 4. presents the indicators of validity in details according to the ICD10 subcategory).

**Table 3:** The sensitivity and positive predictive values of validity for the death certificates (major grouping).

Major disease categories (ICD10 rubric)	True positive*	False Positive*	False negative*	Sensitivity	Positive Predictive value
Neoplasms C00-D48	32	13	3	0.91	0.71
Endocrine and metabolic diseases (E00-E88)	1	4	0	1.00	0.20
Diseases of the nervous system (G00-G98)	2	2	3	0.40	0.50
Diseases of the circulatory system (I00-I99)	69	19	34	0.67	0.78
Diseases of the respiratory system (J00-J98)	3	6	2	0.60	0.33
Diseases of the digestive system (K00-K92)	6	2	2	0.75	0.75
Diseases of the genitourinary system (N00-N98)	0	1	4	0.00	0.00
Transport accidents (V01-X59)	16	3	6	0.73	0.84

*Expressed as number. Categories with less than 5 events were omitted.

**Table 4:** The sensitivity and positive predictive values and other measures of validity for underlying cause of death for the death certificates (major sub categories of ICD10).

Disease sub-category grouping (ICD-10 rubric)	True positive*	False Positive*	False negative*	Sensitivity	Positive Predictive value
*Malignancy (C00-D48)*					
Stomach cancer (C16.0–C16.9)	6	1	0	1.00	0.86
Malignant neoplasm of liver and intrahepatic bile ducts (C22.0–C22.9)	2	1	0	1.00	0.67
Malignant neoplasm of pancreas (C25.0–C22.9)	3	0	0	1.00	1.00
Malignant neoplasm of colon (C18.0–C18.9)	2	0	1	0.67	1.00
Malignant neoplasm of lung (C34.0–C34.9)	2	0	0	1.00	1.00
Malignant neoplasm of bladder (C67.0–C67.9)	2	1	1	0.67	0.67
Malignant neoplasm of brain (C71.0–C71.9)	2	1	1	0.67	0.67
Malignant neoplasm of breast (C50.0–C50.9)	4	0	0	1.00	1.00
*Endocrine, nutritional and metabolic diseases (E00-E99)*					
Diabetes mellitus (E10–E14)	1	3	0	1.00	0.25
*Diseases of the nervous system (G00-G98)*					
Systemic atrophies affecting the central nervous system (G10–G13)	2	0	1	0.67	0.100
*Diseases of the circulatory system I00-I99*					
Ischemic heart diseases (I20–I25)	43	15	32	0.57	0.74
Cerebrovascular diseases (I60–I69)	13	8	10	0.57	0.62
Hypertensive heart disease (I10–I15)	1	1	0	1.00	0.50

*Expressed as number.

Assessing the determinants of a valid cause-of-death assignment, death at age group over 60 compared with under 60 (OR of 2.5 and 95% CI 1.1 5.9) and assigning cause of death by general physician compared with a specialist (OR of 2.3 with 95% CI of 1.1, 5.1) were associated with inaccurate assignment of cause of death. The Hosmer-Lemeshow Chi-square equaled 3.1 which indicated a statistically fitted logistic model. Table 5 present the details of the logistic regression model.

**Table 5:** The result of logistic model detailing determinants of a valid cause-of-death assignment.

Variable	OR	95% CI*	P value
Age (≥60 vs <60)	2.49	1.05–5.92	0.039
Sex (male vs female)	1.31	0.69–2.51	0.411
Place of death (Home vs hospital)	0.96	0.44–2.09	0.915
Assigning cause of death (GP** vs Specialist)	2.30	1.05–5.08	0.040

*Confidence Interval.

**General Physician.

## Discussion

Our study showed that neoplasms were over reported and death due to circulatory system and transport accidents underreported in the death certificate issued in the population of Tehran, indicating different degrees of accuracy for different causal categories. The reported predictive accuracy of underlying cause of death based on pre-mortem information indicates wide variations depending on several factors mainly the true underlying cause of death [14]. While cancers are reported with a high degree of validity, infectious diseases are of low predictive value of accuracy [8], [15]. Our study demonstrated that for the leading causes of deaths especially cancer acceptable degree of validity was observed in death certificates a finding comparable and concordant with other's reports [16]. The reason for high predictive accuracy of death certificate when the underlying causes are cancers is because these morbid conditions are relatively well-characterized helping assignment of the underlying cause of death more accurate compared with a sudden and unobserved morbid condition [5], [17]. Several studies exploring the validity of cancer as underlying cause of death has reported a strong association between a cancer site and its high positive predictive values; cancers of esophagus, stomach, colon has been associated with high degree of being accurately reported as true cause of death in the death certificate by different authors [16], [18]. Our data showed high degree of validity for cancers of specific organ such as stomach and colon. Contrary to findings our study, mortality from cancer has always been complicated with the fact that primary cancers are misclassified with metastatic cancers, resulting in a higher rate of mortality for cancers of lung, and liver [19], [20]. Small number of cases with underlying cause as cancer in our study limits further scrutiny of measuring validity of this major category of diseases.

The other leading cause of death in our study was diseases of circulatory system with a relatively high positive predictive value at the major category of ICD10 grouping and with lower sensitivity and positive predictive value in sub-grouping level of ICD10. The sensitivity and predictive values of diseases of circulatory system in the death certificate has been studied extensively [21], [22], depending on the gold standard being used; when necropsy was used as gold standard very low sensitivity and positive predictive value were reported [9] when the pre-mortem clinico-pathological information are used a better accuracy are reported [14]. In the ICD10 classification system, diseases of circulatory system are sub-categorized into 10 groups of which three group constitute majority of the deaths, the ischemic heart disease, cerebrovascular diseases, and hypertensive heart diseases. The fact that our data showed a underreporting of disease of circulatory system is the result of a major part of death certificate in both, TLGS and original death certificate that were coded as undetermined. Such assignment indicates a poor wording and lack of procedural system in handling the death certificate in the studied population.

The third leading cause of death in our community is injury and traffic accident [23]. An acceptable sensitivity and positive predictive value is reported for this category of death certificates in similar studies as the death circumstance is well defined and involves the legal system and other administrative bodies such as insurances [24].

The pattern of misclassification (an under reporting of circulatory system and transport accident) in our study contrast other studies in which the diseases of circulatory systems are over reported. Such a discrepancy may originate from the fact that a sizeable part of causes declared in the death certificate in our study were coded as undetermined due to lack of quality information for the physician to assign an underlying cause.

The methodology involved in assessment of accuracy of death certificate have been mainly of comparing the reported underlying cause of death certificate with a gold standard. A systematic review looking at the studies evaluating death certificate validity, reported 89% percents of studies used pre-mortem clinicopathological evidence as gold standard compared just to 7% that used autopsy [25]. Our study utilized the resources of a well established cohort study to ascertain cause of death, similar use of information generated in cohort studies has been reported for famous Framingham study in which fifty years of death in the cohort were investigated against the death certificate issued to deceased cohort member [1], [14], [24]. The use of information generated in cohort studies not only can be best fit to study the validity of cause of death, but also, in our view, it could be used as means to truly assess the established procedures in assigning cause of death and coding practice as in the cohort studies the events of the natural history of disease progression can be well documented.

What factors influence the assignment of cause-of-dearth in a death certificate has been a subject of several studies [6], [7], [26]. Apart from true cause of death as a major determinant of cause-of-death assignment, other factors such as age, sex, race, socioeconomic of the deceased or its proxies such as education level have been associated with accuracy of cause-of-death assignment. Our result is concordant with other studies in terms of age of deceased in predicting a true cause of death (the older the more chance of inaccuracy). The fact that being a GP and having two times chance of assigning an inaccurate cause-of-death in our study is again comparable to literature where differential and higher mortality rate have been reported for general practitioners [27], [28]. The fact that more death certificate was issued by GP's compared with specialist signify the need for interventions aiming at systematic training of the GP's in our community for better handling of the death certificate.

Result of our study may be subject to weak external validity as our subject came from a cohort that has been selected based on certain criteria which may make the cohort not a representative of the general population though comparable findings of our study with other studies make this uncertainty less important. In addition, the fact that the COP was unable to determine an underlying cause for 18.2% of cases may have biased our relatively high sensitivity due to the fact that if a underlying cause was determined for this group, the chances was that some of them would be classified to the cancer or circulatory system resulting in lower sensitivity for these groups. Another major limitation of our study was small number of deaths. This resulted in estimation of sensitivity and positive predictive values to suffer from small numbers especially in categories that lower mortality rates are observed.

In summary, the present study shed light on the validity of death certificate in our population helping to assess the reliability of mortality statistics in our population. Further studies are needed to address all aspect of mortality information in order to develop administrative procedures in improving validity of mortality statistic in our community.
